# Recent advances in understanding resistance exercise training-induced skeletal muscle hypertrophy in humans

**DOI:** 10.12688/f1000research.21588.1

**Published:** 2020-02-24

**Authors:** Sophie Joanisse, Changhyun Lim, James McKendry, Jonathan C. Mcleod, Tanner Stokes, Stuart M. Phillips

**Affiliations:** 1 Exercise Metabolism Research Group, Department of Kinesiology, McMaster University, Hamilton, ON, Canada

**Keywords:** resistance exercise, muscle, protein, hypertrophy

## Abstract

Skeletal muscle plays a pivotal role in the maintenance of physical and metabolic health and, critically, mobility. Accordingly, strategies focused on increasing the quality and quantity of skeletal muscle are relevant, and resistance exercise is foundational to the process of functional hypertrophy. Much of our current understanding of skeletal muscle hypertrophy can be attributed to the development and utilization of stable isotopically labeled tracers. We know that resistance exercise and sufficient protein intake act synergistically and provide the most effective stimuli to enhance skeletal muscle mass; however, the molecular intricacies that underpin the tremendous response variability to resistance exercise-induced hypertrophy are complex. The purpose of this review is to discuss recent studies with the aim of shedding light on key regulatory mechanisms that dictate hypertrophic gains in skeletal muscle mass. We also aim to provide a brief up-to-date summary of the recent advances in our understanding of skeletal muscle hypertrophy in response to resistance training in humans.

## Introduction

Skeletal muscle is the organ of locomotion but is also a large contributor to resting energy expenditure ^1^ and is the largest reservoir for post-prandial insulin-stimulated disposal of blood glucose ^2^. Thus, beyond skeletal muscle’s obvious role in locomotion and mobility, its maintenance is critical for metabolic health. Indeed, lower-than-predicted norms of skeletal muscle mass and function are associated with a variety of negative health outcomes such as cardiovascular disease, cancer, and increased risk for disability ^3^. Therefore, concerted efforts to maintain, increase, or regain lost skeletal muscle mass (for example, due to muscle disuse) are of relevance to human health ^4^.

Skeletal muscle exhibits an extraordinary range of phenotypic plasticity in response to changing contractile stimuli. Skeletal muscle hypertrophy can be defined as an increase in muscle axial cross-sectional area (CSA), assessed via magnetic resonance imaging (MRI), computed tomography, ultrasound, and/or biopsies examining muscle fiber CSA (fCSA). Presently, chronic resistance exercise (RE) training (RET) and sufficient dietary protein feeding provide the most effective non-pharmacological strategies to promote skeletal muscle hypertrophy ^5^. Significant attention has been directed towards deciphering the mechanistic underpinnings of what gives rise to skeletal muscle hypertrophy. The purpose of this review is to provide a brief up-to-date narrative on recent advances in our understanding of RET-induced skeletal muscle hypertrophy. It is notable that similar topical reviews have recently been published (see references ^6– 8^), and they should be consulted to obtain other viewpoints on this topic.

## Exogenous versus endogenous variables in determining hypertrophy

Muscle hypertrophy is influenced by factors that can be broadly grouped into two categories: exogenous and endogenous variables. Exogenous factors include RE-related variables (load, reps, time under tension, volume, etc.), diet-related variables such as protein supplementation, energy intake, and consumption of anabolic supplements (i.e. creatine), and administration of anabolic hormones. The hypertrophic response to RET can be augmented marginally via greater-than-recommended protein ingestion, but the response is saturated around self-reported intakes of ~1.6 g protein/kg body mass/day ^5^; however, in resistance-trained individuals, protein intake may need to be greater (~2.0–2.2 g protein/kg body mass/day) to maximize whole-body anabolism ^5, 9^. Specifically, leucine has been repeatedly shown to be the most potent, and possibly exclusively in human skeletal muscle ^10^, amino acid agonist that induces muscle protein synthesis (MPS) ^10– 12^.

Endogenous variables, namely genomic, epigenetic, transcriptomic, and proteomic variables ^13^, are determinants of muscle hypertrophy. Importantly, each of these variables can ultimately be affected by exogenous variables, such as nutrition and RET paradigms, to which they may show differential responses. Extant literature demonstrates that manipulation of some RET variables has, at best, statistically significant but relatively small effects that are for the most part related to greater mechanical work (although this too would have a ceiling) and are most easily outwardly manifested by high(er) degrees of effort ^14^. What is abundantly clear is that transient post-exercise rises in systemic concentrations of various anabolic hormones (testosterone, growth hormone, and insulin-like growth factor 1 [IGF-1]) are unrelated to muscle hypertrophy ^15, 16^.

**Figure 1.**
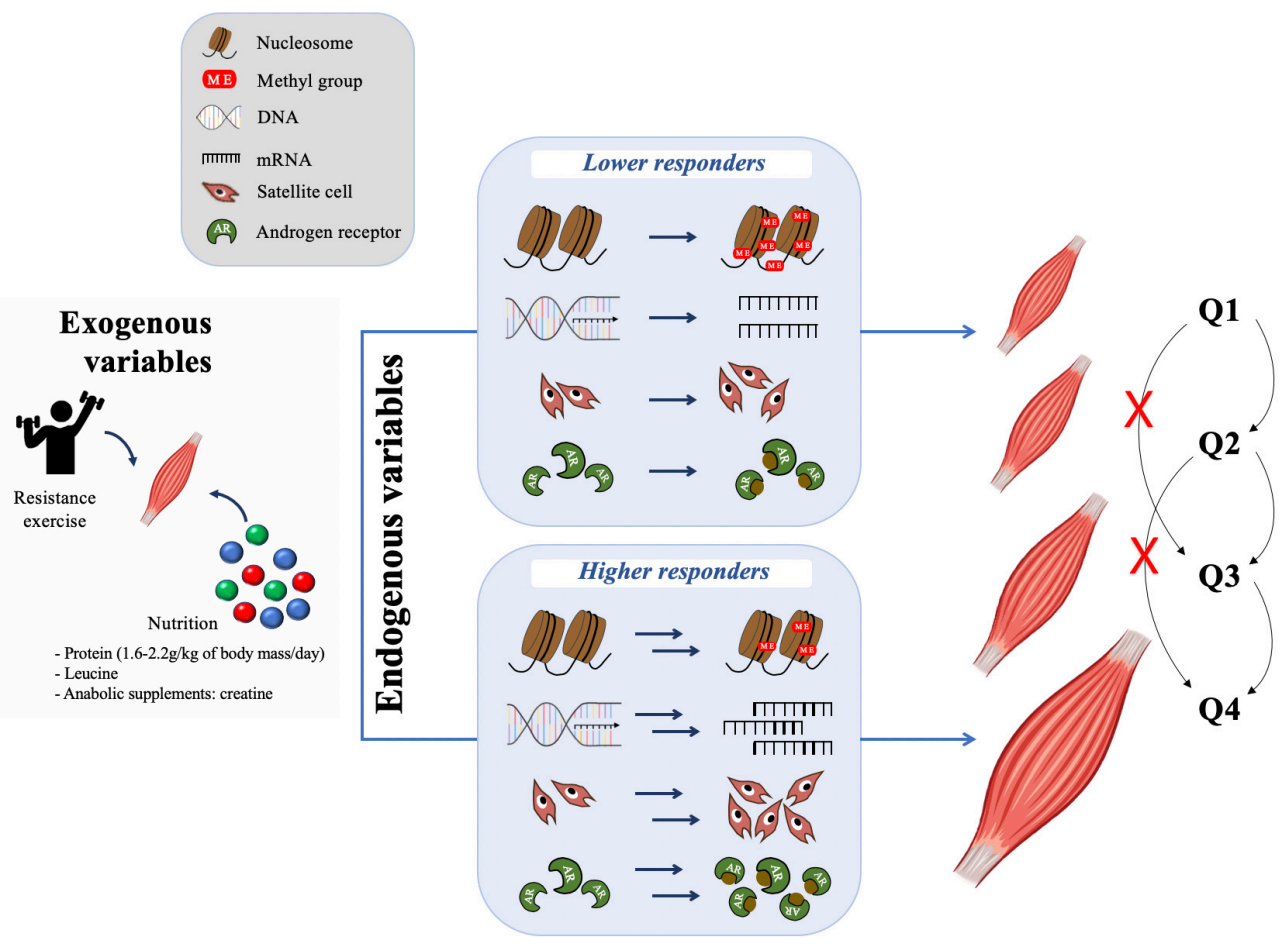
Current understanding of the relationship between exogenous and endogenous variables for skeletal muscle hypertrophy. Appropriate exogenous stimuli are required to modulate endogenous variables related to muscle protein synthesis and induce skeletal muscle hypertrophy. Resistance exercise and nutrition variables such as dietary protein (especially leucine) as well as anabolic supplements are considered to be the most reliable exogenous variables for skeletal muscle hypertrophy. However, the red arrow-headed line and red dotted line illustrate that exogenous variables do not induce skeletal muscle hypertrophy independently of the endogenous variable modulation. Therefore, endogenous variables are affected by exogenous variables, such as modification to histones, transcription factors, satellite cells, and/or androgen receptor content, which are key determinants of skeletal muscle hypertrophy. The blue arrow-headed line describes the exogenous stimuli that must act through endogenous variables, as represented by thin blue lines, to induce skeletal muscle hypertrophy. Furthermore, depending on the extent of the endogenous variables’ response to exogenous stimuli, higher responders may have greater skeletal muscle hypertrophy compared to lower responders.

Although exogenous variables are important, it is becoming more widely appreciated that the endogenous molecular responses to RE are paramount in determining the hypertrophic response. Intramuscular mechanosensitive signaling pathways and extracellular supporting structures (i.e. extracellular matrix and capillaries) appear to play important roles in hypertrophy ^17^. While evidence is equivocal ^18, 19^, our laboratory has demonstrated that individuals exhibiting greater hypertrophy in response to RET appear to have greater androgen receptor content at rest ^16^, and the change in androgen receptor content is positively correlated with increased fCSA following RET ^20^. Moreover, an enhanced satellite cell (SC) proliferation in response to loading ^21^ differentiates higher from lower hypertrophic “responders” to RET. Furthermore, the aforementioned endogenous variables—higher androgen receptor content and augmented SC proliferation—have been reported to be greater in “high” compared to “low” responders to RET ^22– 24^. Stimulation of MPS can also occur owing to increased efficiency of translation, with more mRNA translated per ribosomal unit ^25^, or to increased translational capacity, which occurs by adding more ribosomes to translate existing mRNA. Therefore, ribosomal biogenesis has also been purported as an endogenous variable related to muscle hypertrophy ^6, 26^. This concept is discussed in more detail further in the review. A schematic of these relationships is summarized in Figure 1. A tenet illustrated in this figure is that in response to mechanical loading, there are degrees of hypertrophic response on which people can, but also cannot, improve. Thus, similar to variability in response to any external stimulus, there is a response variability in exercise-induced hypertrophy that is propelled by external variables but predominantly translated into muscle growth through endogenous variables. Clearly, we do not have a complete picture of the loading-induced hypertrophic process, and further research is needed to define the relationship between exogenous variables and their effect on endogenous variables that directly mediate pathways leading to muscle hypertrophy.

## Protein turnover and its role in skeletal muscle hypertrophy

Skeletal muscle hypertrophy occurs as the result of recurrent periods of positive net protein balance (NPB), when the rate of MPS exceeds that of muscle protein breakdown (MPB). In the post-absorptive (i.e. fasted) state, rates of MPB exceed MPS, resulting in a negative NPB ^27^. Importantly, nutrition and contractile activity are potent modulators of MPS and, to a lesser extent, MPB in both trained ^28– 30^ and untrained individuals ^31^. Specifically, in the post-absorptive state, RE stimulates increases in both MPS and MPB, and while MPS is stimulated to a greater extent, NPB remains negative ^30^. Ingestion of dietary protein containing sufficient essential amino acids ^30^, in close temporal proximity to RE, augments MPS and attenuates the exercise-induced increase in MPB. Therefore, only when RE is coupled with protein feeding does NPB become positive, facilitating small periods of muscle protein accrual with RET that sum to yield eventual hypertrophy ^27^.

**Figure 2.**
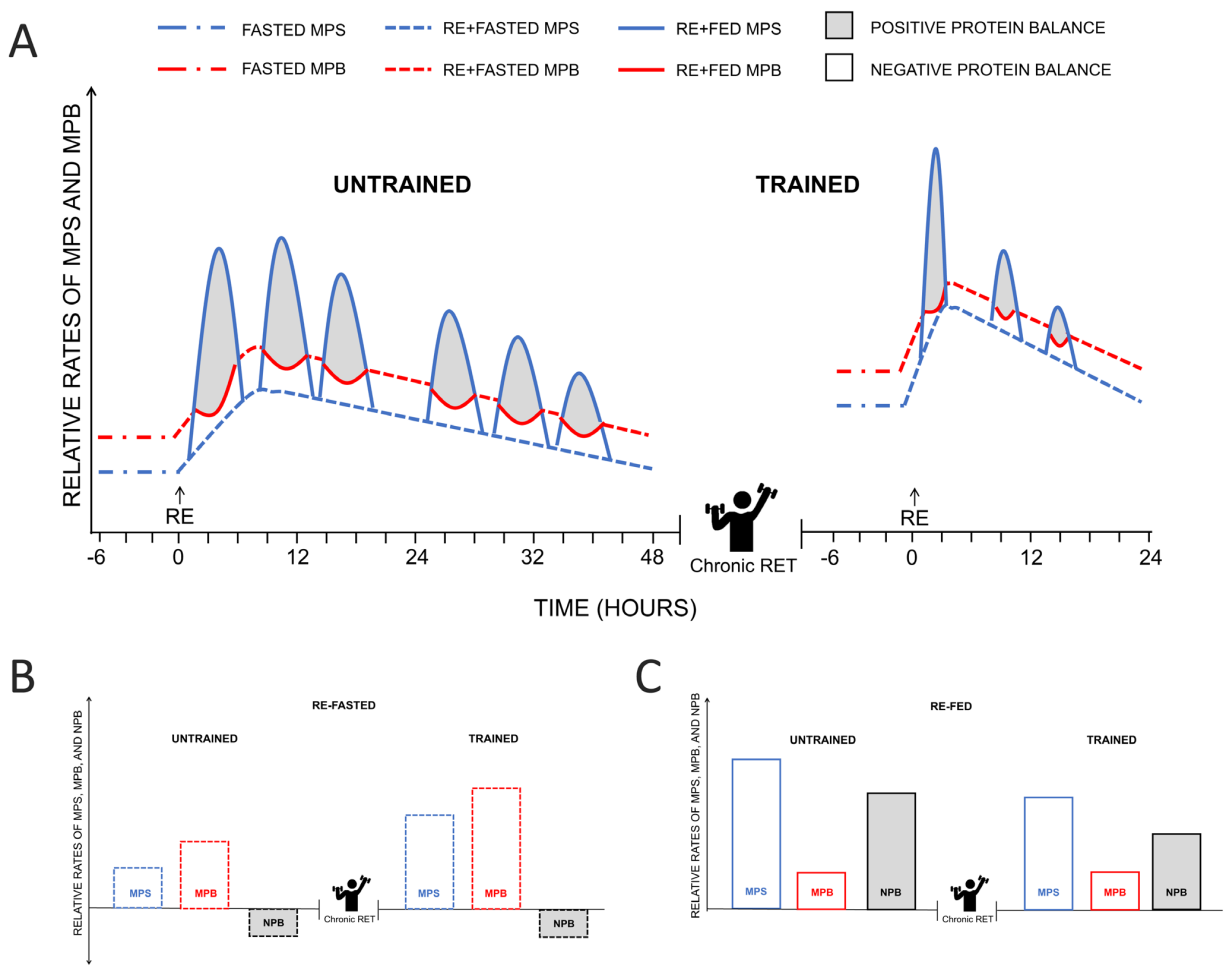
Current understanding of changes in muscle protein turnover with chronic resistance exercise training. Skeletal muscle hypertrophy can occur only under periods of positive protein balance: that is, when relative rates of muscle protein synthesis (MPS) (blue line) exceed that of muscle protein breakdown (MPB) (red line). In the fasted state, rates of MPB exceed those of MPS, resulting in a negative net protein balance (NPB). Compared to untrained individuals ( **A**), trained individuals ( **B**) display higher fasted rates of MPS; however, protein balance remains negative because of the concomitant elevation of MPB in the trained state. Regardless of training status, nutritional and contractile stimuli are potent regulators of MPS and, to a lesser extent, MPB. Resistance exercise (RE) stimulates increases in both MPS and MPB, and NPB remains negative. Ingestion of dietary protein—in particular, essential amino acids—in close temporal proximity to RE augments MPS and attenuates the exercise-induced increase in MPB, resulting in a temporary state of positive protein balance. Chronic RE training (RET) modulates the time course of the increase in MPS following a bout of RE. Specifically, the initial increase in MPS following a bout of RE is less pronounced in the untrained state than in the trained state; however, it is longer lived and peaks later in the untrained than the trained state. MPS, MPB, and NPB during periods of ( **B**) RE+Fasted and ( **C**) RE+Fed in the untrained and trained state.

Changes in post-absorptive MPS are modified with RET (for review, see 32). Elevated post-absorptive MPS has been proposed as a primary contributor to muscle hypertrophy with RET (>6 weeks) ^6^. Indeed, early observations in humans show that post-absorptive MPS is elevated in the trained state ^30, 33, 34^. However, identical to what is seen in untrained individuals, NPB in the post-absorptive state is always negative because of a concomitant elevation of MPB in trained individuals ^30, 32^. Thus, the trained state is demarked by an enhanced overall rate of protein turnover—elevated rates of MPS and MPB—that favors only net protein accretion, as demonstrated multiple times ^26, 32, 35^, in the fed state. The elevation in MPB in the trained state is also supported by molecular evidence ^36^. Acute intermittent elevations in MPS in response to, and with persistent practice of, RE in combination with sufficient protein feeding are undeniably the major drivers of muscle protein accretion and skeletal muscle hypertrophy ^37^. We speculate that the overall increased protein turnover (as a result of cumulative greater acute periods of positive NPB) observed with chronic RET is advantageous and is reflective of a general increase in turnover of muscle proteins (i.e. upregulation of MPS and MPB) that favors efficient remodeling of protein that leads to a gradual muscle protein accrual manifested as hypertrophy ^32^; these concepts are depicted schematically in Figure 2.

At the molecular level, RE and protein feeding increase MPS through mechanistic target of rapamycin complex 1 (mTORC1)-dependent ^38^ and -independent ^38, 39^ mechanisms. Typically, mTORC1 phosphorylation activates several downstream kinases, augmenting translational efficiency (i.e. an increase in the rate of translation of mRNA by a constant pool of ribosomes) and, with RET, translational capacity (i.e. total number of available ribosomes) ^26, 38^. Recently, it has been suggested that increased translational capacity is central to changes in post-absorptive MPS with chronic RET ^6^. Several groups have demonstrated that chronic RET results in increased total RNA ^19, 40– 42^ and ribosomal RNA (rRNA) content ^24, 40^ in addition to increases in regulators of rRNA synthesis ^24, 40– 42^. In contrast, other groups reported a reduction in biomarkers of ribosomal biogenesis ^43^ or no change following 12 weeks of RET ^19^. Increases in RNA content—following 16 weeks ^44, 45^ and 6 weeks ^18^ of RET—were similar between individuals showing either no change (i.e. “non/low responders”) or an extreme increase (i.e. “extreme/high responders”) in vastus lateralis muscle fCSA. In contrast, Stec and colleagues ^41^ reported that only “extreme” responders to 4 weeks of RET had increases in total RNA and rRNA content. Conflicting results may be attributed to differences in participant characteristics, experimental design, and analytical techniques ^26^; however, current evidence does not demonstrate a clear connection between translational capacity and skeletal muscle hypertrophy in humans ^37^. We hypothesize that early on in a RET program, ribosomal capacity may be increased as a general response to a need for greater rates of global protein synthesis ^46^. However, with persistent practice of RET once protein synthetic responses and transcriptional programs become “refined” and more specific to the stimulus of RET ^34^—as well as being shorter in duration ^32^—further increasing ribosomal capacity is not required and would either stabilize ^40, 42^ or possibly decline ^43, 47^. This thesis would underpin why early during a RET bout a very short-term MPS response does not align well with eventual hypertrophy ^48^, but this is not the case with further RET where MPS shares common variance with hypertrophy ^46^. It should also be noted that the stabilization of ribosomal capacity following chronic RET ^40, 42^ does not indicate a loss of muscle ribosomes *per se*; instead, this likely reflects a dilution of the ribosomal capacity by larger, hypertrophied myofibers.

Understanding changes in translational capacity with RET is limited owing to a number of methodological constraints. Specifically, the study of ribosomal biogenesis relies heavily on static measures (i.e. immunoblotting and quantifying total RNA content and assuming rRNA content is responsible), and traditional stable isotope tracer investigations provide insight into only acute (i.e. hours) metabolic fluctuations ^49^. Recent advances in mass spectrometry techniques have led to the reintroduction of deuterium oxide (D _2_O) ^50, 51^, which enables the assessment of metabolic flux in response to a variety of stimuli, such as skeletal muscle loading ^11, 12, 42, 46^, unloading ^52– 54^, and feeding ^28, 42, 46^ under longer-term, “free-living” conditions (i.e. integrated over days to weeks). Brook and colleagues ^50^ recently validated the use of D _2_O in monitoring the synthesis of ribonucleotides, providing the first dynamic measure of RNA synthesis in human skeletal muscle in response to RET. Of particular note in this study, RNA synthesis was increased above basal rates over the 0–6-week period with continuous RET ^50^. Importantly, myofibrillar MPS in these individuals was not significantly increased above basal levels during this period ^42^, showing a discordance between translational capacity and MPS with long-term muscle adaptations. Future studies incorporating dynamic measures of RNA synthesis and integrated rates of MPS in concert with omic-level measurements should provide a platform to elucidate the relative contribution, and time-course, of translational efficiency and capacity to changes in MPS and hypertrophy in response to chronic RET.

## Omic-based science and skeletal muscle hypertrophy

Our present mechanistic understanding of muscle hypertrophy has largely been informed by the use of “targeted” analytical approaches providing static snapshots (i.e. qPCR and immunoblotting). However, the increased usage of “omic” technologies can offer an unbiased and integrative understanding of the processes regulating muscle hypertrophy. Proteomic profiling has tremendous potential to advance our understanding of muscle growth; however, it is currently constrained by a relatively limited coverage of highly abundant proteins in the proteome versus a far larger coverage of RNA: <500 proteins reliably detected ^55, 56^ versus ~30,000 RNA species ^55^. This low protein:RNA ratio results in an incomplete understanding of downstream ontology/pathway analyses ^57^ but could also mask the important role of less-abundant regulatory proteins in muscle hypertrophy (i.e. signaling molecules ^57^ or integrin receptors ^58^). It is possible to circumvent these limitations by studying the expressed RNA complement of the cell (via transcriptomics) or translatome of the cell (via polysomal RNA and transcriptomics), given the close association between mRNA and protein abundance under most conditions ^59, 60^ and, in particular, the global translatome in skeletal muscle ^61, 62^.

Early applications of transcriptomics have shown that older adults, and lower hypertrophic responders in general ^63^, express a pro-inflammatory gene profile at rest and respond to an acute bout of RE with an exaggerated inflammatory response ^64^, linking inflammation with an attenuated muscle growth response to RET. Elderly adults also have an elevated expression of p21 ^65^, a cell cycle inhibitor that affects SC proliferation ^66^ and may therefore impair muscle growth following RET ^21^. In contrast, higher hypertrophy responders to RET express higher levels of several well-known growth and remodeling genes prior to training compared to lower responders, which is suggestive of a “primed” basal state of protein turnover ^63^. Higher RET responders also express greater levels of oxidative, angiogenic, and extracellular matrix remodeling genes after RET ^65, 67^. Two noteworthy yet ill-characterized genes that are also upregulated in high responders in the basal state include *NAP1L1* and *DGKZ* ^63^, which encode a nucleosome-associated protein and diacylglycerol kinase zeta (DGKζ), respectively. The protein encoded by *NAP1L1* controls chromatin compaction but has also been shown to bind to and regulate the nuclear-cytoplasmic shuttling of DGKζ ^68^. Importantly, DGKζ was shown recently to play a pivotal role in mechanical overload-induced muscle hypertrophy in rodents, but only if the nuclear localization signal of DGKζ was intact ^69^. While the nature of this interaction in humans warrants further investigation, the example attests to the hypothesis-generating power of transcriptome profiling and its inherent potential for biological discovery.

An ongoing challenge in transcriptomics is the use of gene ontology (i.e. DAVID ^70^) and network analytical tools (ingenuity pathway analysis [IPA] ^71^), which are commonly used to uncover functional relationships from large lists of RET-regulated genes. These tools rely on the function(s) of a gene product being known ^56^. However, data-driven networks (DDNs) are networks constructed on the basis of experimentally derived gene co-expression similarities, without *a priori* knowledge of gene function. Clarke and colleagues ^72^ used a DDN approach to construct gene networks from pre- and post-muscle transcriptome samples obtained from the HERITAGE study ^73^ (endurance-based training) and identified *EIF6* as an exercise-responsive highly interconnected “hub” gene. EIF6 was therefore predicted, on the basis of being highly connected to other regulated genes, to play an important role in the adaptation to endurance training. Indeed, subsequent development of a mutant EIF6 murine model was shown to affect many of the same signaling pathways predicted by the HERITAGE study ^72, 73^ that affect phenotype. Greater use of DDNs and network modeling could be applied to the study of muscle hypertrophy with RET with, we propose, great potential.

## SCs and their role in RET-induced hypertrophy

In humans, increases in muscle fiber size are commonly reported with a concomitant increase in the number of myonuclei ^74^, an observation that lends credence to the myonuclear domain theory of muscle growth ^75^. This theory suggests that each myonucleus governs a set volume within the muscle fiber and, when the ceiling of the muscle fiber volume is reached, the transcriptional capacity of an existing myonucleus is reached and new myonuclei must be added to maintain (or re-establish) transcriptional control over a defined myonuclear domain. Skeletal muscle is a post-mitotic tissue; therefore, the addition of new myonuclei must come from a new source, which occurs via donation from skeletal muscle stem cells, i.e. SCs.

Activation of SCs occurs following various stimuli such as injury, damage, and exercise. Once activated, SCs progress from proliferation to terminal differentiation, eventually fusing and donating their nuclei to existing myofibers, a process termed the myogenic program. Although common dogma had long associated SCs with skeletal muscle hypertrophy ^76, 77^, this concept has recently been challenged. McCarthy and colleagues ^78^ were the first to use the Pax7-DTA mouse strain that results in conditional SC ablation to demonstrate that significant overload-induced hypertrophy, via synergist ablation, can occur in SC-depleted rodent skeletal muscle. The same group reinforced these findings using hind-limb suspension, to induce atrophy, followed by reloading and regrowth of muscle which was not affected by SC depletion, in the Pax7-DTA mouse ^79^. Importantly, while interesting, these results highlight that SCs are not necessary for hypertrophy in short-term extreme models of hypertrophy but do not address the question of whether SCs are involved in a more physiologically relevant hypertrophic situation (i.e. following RET). This notion was further challenged by a study from Egner and colleagues ^80^, in which they describe impaired hypertrophy with 2 weeks of overload, via synergist ablation, using the same Pax7-DTA mouse strain ^78, 79^. Further to this, work by Murach and colleagues ^81^ demonstrated that myonuclear accretion via the SC is necessary to support overload-induced hypertrophy in younger growing mice, highlighting that the requirement of SCs to support hypertrophy is affected by age. Notably, the extent of hypertrophy is attenuated following 8 (versus 2) weeks of overload-induced hypertrophy in Pax7-DTA mice ^82^, suggesting that SCs are involved in muscle growth. Importantly, the researchers described an accumulation of the extracellular matrix in SC-depleted mice following 8 weeks of overload, which resulted in the impaired hypertrophic response ^82^. These data suggest that SCs are able to support muscle growth not only by fusing to existing fibers resulting in myonuclear accretion but also by their interaction with other cell types to regulate the extracellular matrix deposition ^83^. Although work in rodent models has been essential in providing insight into the basic cellular and molecular mechanisms that result in muscle hypertrophy, these results cannot always easily be translated to humans. For example, cerebral palsy, a developmental motor disorder characterized by a reduction in muscle fiber size, is also associated with a reduction in SC content ^84, 85^, and it is postulated that the reduction in SC content may contribute to the impairment in muscle growth ^86^. For obvious reasons, it isn’t possible to study the effects of SC depletion in humans, and the observation of SCs in a human model with a reduced (although not ablated) SC content is often confounded by the presence of chronic disease, where factors other than SC content may contribute to the inability of muscle to hypertrophy.

Importantly, the majority of evidence stemming from human studies has implicated a role for SCs in contributing to increases in muscle fiber size. Several studies have described a positive relationship between muscle fiber size and number of myonuclei in human muscle ^19, 21, 47, 87– 92^. In addition, studies have also described an increase in myonuclear number with training-induced fiber hypertrophy concomitant with an increase in SC content ^80, 87– 90^. It is, however, important to note that several groups have reported an increase in fCSA without an increase in SC/myonuclear content ^92– 94^. This may be due to several factors, one of which is the ability of existing myonuclei to increase their transcriptional capacity to support the increase in muscle fiber size ^95^.

Interestingly, individuals classified as “extreme” (hypertrophy) responders to RET had greater basal SC content compared to “lower” and “moderate” responders, which translated to a greater expansion of the SC pool with training and was accompanied by an increase in myonuclear content; however, the myonuclear domain also increased ^21^. Thus, similar to transcriptional observations, the basal characteristics of skeletal muscle (i.e. SC content) may play a role in response plasticity to hypertrophic stimulus. Congruent with previous work ^21^, we demonstrated that the acute SC response to a bout of unaccustomed RE is related to the increase in quadriceps volume observed following training ^87^. Although SCs likely contribute to hypertrophic adaptation via myonuclear accretion, it is important to recognize the ability of resident myonuclei to respond to varying stimuli such as RET and their inherent ability to support growth. The concept of muscle “memory”, manifested through possible epigenetic changes, is also likely an important contributor to the ability of skeletal muscle to hypertrophy. Seaborne and colleagues ^96^ demonstrated that prior RE-induced hypertrophy enhanced the subsequent response to a bout of resistance training, following a period of detraining, which may be a consequence of the widespread hypomethylation incurred during the first adaptive response. Together, the evidence in humans reporting an increase in muscle fiber size with a concomitant increase in myonuclei ^19, 21, 47, 87– 92^ highlights that SCs likely play a role in mediating skeletal muscle hypertrophy. However, as shown by Kirby and colleagues ^95^, using a time-course experiment following synergist ablation in the Pax7-DTA mouse model, the ability of existing resident myonuclei to support periods of fiber growth cannot be disregarded.

## Conclusion and future directions

Skeletal muscle plays an indispensable role in an array of mechanical and metabolic functions ^97^. Typically, as we age, the quantity and quality of skeletal muscle deteriorates owing to the infiltration of non-muscle tissue including adipose and connective tissue ^98^. Therefore, concerted efforts to increase and maintain skeletal muscle mass should be made by a range of individuals spanning from those striving to improve athletic performance to those focused on extending the healthspan. RE and dietary protein act synergistically and, at present, provide the most effective strategy to augment skeletal muscle mass ^37^. Skeletal muscle hypertrophy is a complex process with multiple regulatory gene/protein hubs that have recently received significant attention in helping to decipher the mechanistic underpinnings that dictate the skeletal muscle adaptive response. As a result, a number of exogenous factors that influence endogenous pathways have been identified to play an important role in skeletal muscle hypertrophy.

MPS is the principal locus of control that influences muscle protein accretion in response to anabolic stimuli, as opposed to MPB ^28– 31^. However, the relative contribution of increased translational efficiency and translational capacity in affecting hypertrophy remains unclear. Intermittent elevations in rates of MPS in response to exogenous stimuli (i.e. RE and protein nutrition) drive muscle hypertrophy ^28– 31^. Nevertheless, research focused on translational capacity is in its infancy, and the proposed importance ^6^ of ribosomal biogenesis has yet to be confirmed.

What is clearly evident is that muscle hypertrophy is a multi-faceted process. However, targeted approaches that probe specific genes and proteins will provide only an incomplete picture of muscle growth. Unbiased, global “omic” technologies have the potential to provide a more comprehensive understanding of the underlying prerequisites for muscle growth but have inherent limitations that need to be considered.

Myonuclear accretion, due to a loading stimulus, is a means by which the transcriptional capacity of the skeletal muscle may be increased. The addition of new myonuclei is due to the activation and subsequent fusion of SCs to muscle fibers, and substantial evidence shows a role for SCs in muscle hypertrophy in humans. Although this is speculative, we hypothesize that resident myonuclei likely possess the ability, possibly through epigenetic modification, to increase transcriptional capacity to a certain extent, ultimately supporting muscle growth.

Although significant progress has been made, considerable work remains to be done in order to deepen our understanding of the processes that govern RET-induced muscle hypertrophy. Future studies incorporating dynamic measures of RNA synthesis, integrated rates of MPS, and SC/myonuclei assessments in concert with “omic” technologies and DDNs will provide a platform to elucidate the relative contribution, and time-course, of translational efficiency and capacity to changes in MPS and hypertrophy in response to chronic RET.
